# The difference of gut microbiome in different biliary diseases in infant before operation and the changes after operation

**DOI:** 10.1186/s12887-022-03570-1

**Published:** 2022-08-24

**Authors:** Xinhe Sun, Yaoyao Cai, Wenwen Dai, Weiwei Jiang, Weibing Tang

**Affiliations:** 1 Department of Pediatric Surgery, Children’s Hospital of Nanjing Medical University, Nanjing, China; 2 Department of Pediatric Surgery, Yancheng Maternity and Child Health Care Hospital, Yancheng, China

**Keywords:** Biliary atresia, Cholestatic, Gut microbiome, Intestinal flora, 16S rDNA

## Abstract

**Background:**

Evidence supports an association between cholestatic liver disease and changes in microbiome composition. Nevertheless, the identification of this special type of biliary atresia from non-biliary atresia cholestasis is still a major clinical difficulty. The purpose of this study is to compare the differences in the composition of gut microbiome between infants with biliary atresia and infant with non-biliary atrestic cholestasis, to find new ways to identify and diagnose these two diseases early, to understand the influence of the presence or absence of bile on the composition of the gut microbiome in infants with cholestasis.

**Methods:**

Using 16S rDNA gene sequencing technology to analyze the intestinal flora of the participants.

**Results:**

In terms of diversity, there is an obvious structural separation in the intestinal microbiota of the BA group and the CD group, and this structural separation also exists in the comparison between the two groups before surgery. Taxonomic analysis demonstrated that the two groups showed an increase in Proteobacteria and Firmicutes before surgery, and the relative abundance of potential pathogens such as Shigella, Streptococcus, Klebsiella, etc. increased, potential probiotics such as Bifidobacteria and Lactobacillus decreased, but the relative abundance of each genus was different between groups. It was found that Enterococcus, Ralstonia, Nitriliruptoraceae, etc. were differentially enriched in the BA group, the CD group are mainly enriched in Veillonella, Clostridium_sensu_stricto_1 and Lactobacillus. Functional analysis of the groups showed that the BA group mainly focused on the processes of energy release processes, and the CD group mainly focused on the biosynthesis of amino-acids to consume energy.

**Conclusions:**

The composition of intestinal flora is different between biliary atresia and non-biliary atretic cholestasis. Enterococcus, Ralstonia, etc. may become biomarkers for the identification and diagnosis of both.

## Background

Infant cholestatic jaundice is a complicated and easily misdiagnosed disease. It refers to the decrease or obstruction of the flow of bile after the formation of infancy, causing the bile substances that originally to pass through the common bile duct and finally enter the intestinal cavity remain in the liver [1]. The incidence of the disease is about 1/2500, and the incidence is significantly increased in premature infants. Common clinical manifestations include jaundice, large liver, hard texture, lighter feces, darker urine, and itching [2]. The neonatal period is prone to misdiagnosed cholestasis as physiological jaundice, thus delaying the diagnosis of the disease. In most infants, long-term physiologic jaundice is due to benign cases of breast milk jaundice, but also a small proportion of these infants are caused by cholestasis and require timely diagnosis and treatment [3]. In the first few months after birth, the most common cause of cholestatic jaundice is biliary atresia (BA), followed by progressive familial intrahepatic cholestasis (PFIC), premature birth, metabolic and endocrine diseases, and Alagille syndrome (AS), infectious diseases, etc. [4].

Biliary atresia is a progressive fibrosis obstructive cholangiopathy involving the extrahepatic and intrahepatic biliary tract system [5]. The disease eventually progresses to biliary cirrhosis with portal hypertension, end-stage liver disease, and death, typically within the first 2 years of life if left untreated. The etiology of biliary atresia is unknown, but it is thought to involve cholangiocyte morphogenesis disorders, viral infection, toxins, chronic inflammation, or autoimmune-mediated bile duct injury [6]. The current standard procedure for the clinical treatment of biliary atresia is the Kasai portoenterostomy(KPE), which creates the Rouxen-Y intestine by removing the closed extrahepatic bile duct to reestablish the bile flow to the intestine. If KPE is performed within 60 days after birth, 70% of patients will establish bile outflow; After 90 days, less than 25% of patients will have bile flow [7]. Therefore, a timely diagnosis of BA is very important to optimize the prognosis of Kasai surgery. At present, the clinical distinction between biliary atresia and non-biliary atretic cholestasis (hereinafter referred to as cholestasis) is mainly through serological markers (such as direct bilirubin, bile acid, etc.), ultrasonography, radionuclide hepatic radiography, etc., and the diagnosis rate is not high. New biological markers have also been actively sought to improve early diagnosis rates.

Gut microbiome have been found to be closely associated with multiple diseases such as obesity [8], osteoporosis [9], diabetes [10], Alzheimer's disease [11], gastrointestinal cancer [12], and inflammatory bowel disease [13]. More importantly, it has been shown that gut microbiome is involved in the occurrence and development of hepatobiliary diseases. Juanola et al. [14] found that the absence of the gut microbiome leads to increased bile infarct area and reduced ductal response in acute cholestasis. Isaacs et al. [15] found that macrophages contribute to promoting intestinal permeability and to altered microbiome composition through activation of the inflammasome, overall leading to increased endotoxin flux into the cholestatic liver. Wang et al. [16] found lower intestinal microbiota diversity and significant structural separation in infant with biliary atresia as compared with healthy controls. The dysbiosis of intestinal microbiota was further aggravated after the Kasai surgery in the BA infants, by use of broad-spectrum antibiotics, with significantly lower microbial diversity and larger distance from the healthy cohort. Nonetheless, the differences in the gut microbiome in biliary atresia and cholestasis remain unclear.

This study is to collect preoperative and postoperative feces from infant with biliary atresia and cholestasis, detect the changes of intestinal microbiota through 16S rDNA technology, explore new ideas in the identification and diagnosis of the two diseases, find specific potential pathogens of biliary atresia, and understand the influence of the presence or absence of bile on the composition of the gut microbiome in biliary diseases.

## Methods

### Study objects

From November 2019 to September 2020 at Children's Hospital of Nanjing Medical University, 25 infants meeting the inclusion exclusion criteria were collected and classified into cholestasis (CD group) and biliary atresia (BA group) according to the postoperative diagnosis (Table 1).

**Table 1:** Background information of participants

	BA-group(*n* = 17)	CD-group(*n* = 8)	*P*-value
**Gender**			0.613
Male[n(%)]	8(47.1%)	4(50.0%)	
Female[n(%)]	9(52.9%)	4(50.0%)	
**Age of surgery (day,Mean ± SD)**	48.35 ± 16.07	56.38 ± 13.64	0.761
**Feeding pattern**			0.734
Breastfeeding[n(%)]	7(41.2%)	2(25.0%)	
Formula feeding[n(%)]	5(29.4%)	3(37.5%)	
Mixed[n(%)]	5(29.4%)	3(37.5%)	
**Delivery pattern**			0.387
Natural delivery[n(%)]	9(52.9%)	3(37.5%)	
Cesarean section[n(%)]	8(47.1%)	5(62.5%)	

Supplement: All patients included in this study were term infants

The ± indicates the variable unit

### Inclusion and exclusion criteria

Inclusion criteria: 1. Infants younger than 90 days of surgery; 2. Infants who were diagnosed as cholestasis before the operation, and were confirmed to be biliary atresia or biliary obstruction through laparoscopic exploration or cholangiography during the operation, and eventually underwent Kasai operation or biliary irrigation; 3. Infants without other diseases of the digestive system such as diarrhea, bacillary dysentery, enteritis, etc.; 4. Compared with the standard stool color chart, infants whose feces has turned yellow or green 1 week after surgery; 5. Infants whose direct bilirubin decreased by more than 50% at the time of reexamination in the first month after the operation compared with that at the time of discharge.

Exclusion criteria: 1. Infants whose pregnant mothers have chronic liver diseases, diabetes, cardiopulmonary diseases and other chronic underlying diseases; 2. Infants whose pregnant mother has a history of special medication, smoking, drug or alcohol abuse; 3. Infants whose mothers have long-term use of drugs and/or potential probiotics during pregnancy; 4. Infants with severe comorbidities such as liver cirrhosis and hepatic encephalopathy.

### Sample collection and preservation

Fresh feces, corresponding liver function index (ALT, AST), bilirubin (TBIL, DBIL) were collected in the BA and CD group 1 day before surgery, 1 week after surgery and 1 month after discharge. The feces was uniformly kept in the -80℃ refrigerator.

### 16S rDNA sequencing

The 16S rDNA sequencing and data analysis of the intestinal microbiota were completed by Hangzhou Lianchuan Biological Co., Ltd. The steps are described below.

1. DNA extractions: DNA from different samples was extracted using the E.Z.N.A. ®Stool DNA Kit(D4015, Omega, Inc., USA) according to manufacturer’s instructions. The reagent which was designed to uncover DNA from trace amounts of sample has been shown to be effective for the preparation of DNA of most bacteria. Nuclear-free water was used for blank. DNA extraction quality was detected by agarose gel electrophoresis, and DNA was quantified by UV spectrophotometer (The total quality of DNA extraction is not less than 50 ng). The total DNA was eluted in 50 μL of Elution buffer and storedat -80 °C until measurement in the PCR by LC-Bio Technology Co., Ltd, Hang Zhou, Zhejiang Province, China.
2. PCR amplification and 16S rDNA sequencing:

**Table:** 

Region	Primers
V3-V4 [17]	341F (5'-CCTACGGGNGGCWGCAG-3') 805R(5'-GACTACHVGGGTATCTAATCC-3')
Archae [18]	F(5'-GYGCASCAGKCGMGAAW-3') R(5'-GGACTACHVGGGTWTCTAAT-3')
V4 [19]	515F(5'-GTGYCAGCMGCCGCGGTAA-3') 806R (5'- GGACTACHVGGGTWTCTAAT-3')
V4-V5	F(5'-GTGCCAGCMGCCGCGG-3') R(5'-CCGTCAATTCMTTTRAGTTT-3')

The 5' ends of the primers were tagged with specific barcods per sample and sequencing universal primers. PCR amplification was performed in a total volume of 25 μL reaction mixture containing 25 ng of template DNA, 12.5 μL PCR Premix, 2.5 μL of each primer, and PCR-grade water to adjust the volume. The PCR conditions to amplify the prokaryotic 16S fragments consisted of an initial denaturation at 98 ℃ for 30 seconds; 32cycles of denaturation at 98 ℃ for 10 seconds, annealing at 54℃ for 30 seconds, and extension at 72 ℃ for 45 seconds; and then final extension at 72 ℃ for 10 minutes. The PCR products were confirmed with 2% agarose gel electrophoresis. Throughout the DNA extraction process, ultrapure water, instead of a sample solution, was used to exclude the possibility of false-positive PCR results as a negative control. The PCR products were purifyied by AMPure XT beads (Beckman Coulter Genomics, Danvers, MA, USA) and quantified by Qubit( Invitrogen, USA). The amplicon pools were prepared for sequencing and the size and quantity of the amplicon library were assessed on Agilent 2100 Bioanalyzer (Agilent, USA) and with the Library Quantification Kit for Illumina (Kapa Biosciences, Woburn, MA, USA), respectively. The libraries were sequenced on NovaSeq PE250 platform.

### Data analysis

Samples were sequenced on an Illumina NovaSeq platform according to the manufacturer's recommendations, provided by LC-Bio. Paired-end reads was assigned to samples based on their unique barcode and truncated by cutting off the barcode and primer sequence. Paired-end reads were merged using FLASH. Quality filtering on the raw reads were performed under specific filtering conditions to obtain the high-quality clean tags according to the fqtrim(v0.94). Chimeric sequences were filtered using Vsearch software (v2.3.4). After dereplication using DADA2 [20], we obtained feature table and feature sequence. Alpha diversity and beta diversity were calculated by normalized to the same sequences randomly. Then according to SILVA(release 132) classifier, feature abundance was normalized using relative abundance of each sample. Alpha diversity is applied in analyzing complexity of species diversity for a sample through 5 indices, including Chao1, Observed species, Goods coverage, Shannon, Simpson, and all this indices in our samples were calculated with QIIME2 [21]. Beta diversity were calculated by QIIME2, the graphs were drew by R package. Blast was used for sequence alignment, and the feature sequences were annotated with SILVA database for each representative sequence. Other diagrams were implemented using the R package(v3.5.2).

### Statistical method

Clinical characteristics were assessed by the Student's t-test. Statistical analysis was carried out using the GraphPad Prism 8. Comparison between the two groups was performed using the Wilcoxon-test test. Multiple comparisons were performed using the one-way ANOVA and Kruskal–Wallis tests. The correlation model was performed using the sparcc. Significant differences were analyzed using LEfSe (LDA Effect Size) analysis, and statistical methods such as Wilcoxon test, Kruskal–Wallis test and linear discriminant analysis (LDA) were used to find species with significant differences between groups. Prediction of flora function was carried out by PICRUSt2 [22], and then STAMP [23] differences between groups were analyzed by t-test. Data are reported as mean ± SD. The difference was statistically significant at *p* < 0.05.

### Ethics

This study was approved by the Ethics Committee of the Children's Hospital of Nanjing Medical University. All parents of the infants provided written informed consent and voluntarily enrolled their infants in scientific research investigations.

## Results

### Sample characteristics

A total of 25 infants were included in this study, and their feces samples and clinical indicators were collected (Tables 2, 3 and 4). Of these, 17 infants were diagnosed with biliary atresia and 8 infants were diagnosed with cholestasis. At the postoperative review, 16 infants in the BA group recovered well, the jaundice subsided, and the stool color was normal. However, due to various reasons, only fresh feces was collected from 4 infants. All 8 infants in the CD group recovered well, and fresh feces were collected from 3 infants.

**Table 2:** Preoperative clinical indicators

	BA-1	CD-1	*P*-value
TBIL (3.4–17.1 μmol/L)	167.19 ± 44.98	164.95 ± 90.76	0.284
DBIL (0–6.8 μmol/L)	136.16 ± 40.36	129.87 ± 66.01	0.467
ALT (0–41 U/L)	153.29 ± 86.07	193.25 ± 135.61	0.251
AST (0–40 U/L)	220.53 ± 106.44	199.88 ± 119.24	0.880

**Table 3:** Clinical indicators one week after operation

	BA-2	CD-2	*P*-value
TBIL (3.4–17.1 μmol/L)	106.18 ± 39.03	159.92 ± 110.78	0.011
DBIL (0–6.8 μmol/L)	76.26 ± 28.63	130.81 ± 92.76	0.008
ALT (0–41 U/L)	151.94 ± 58.69	219.94 ± 158.02	0.046
AST (0–40 U/L)	164.18 ± 64.28	183.25 ± 90.16	0.278

**Table 4:** Clinical indicators one month after surgery

	BA-3	CD-3	*P*-value
TBIL (3.4–17.1 μmol/L)	30.86 ± 14.72	29.77 ± 19.97	0.166
DBIL (0–6.8 μmol/L)	20.97 ± 11.81	16.20 ± 10.85	0.488
ALT (0–41 U/L)	104.06 ± 58.88	83.00 ± 47.83	0.945
AST (0–40 U/L)	95.47 ± 35.83	78.25 ± 39.37	0.996

Tables 2, 3, 4 Clinical indicators of participants before and after surgery

*TBIL* Total bilirubin, *DBIL* Direct bilirubin, *ALT* Serum alanine aminotransferase, *AST* Aspartate aminotransferase

The ± indicates the variable unit

### Species diversity

**Fig. 1:**
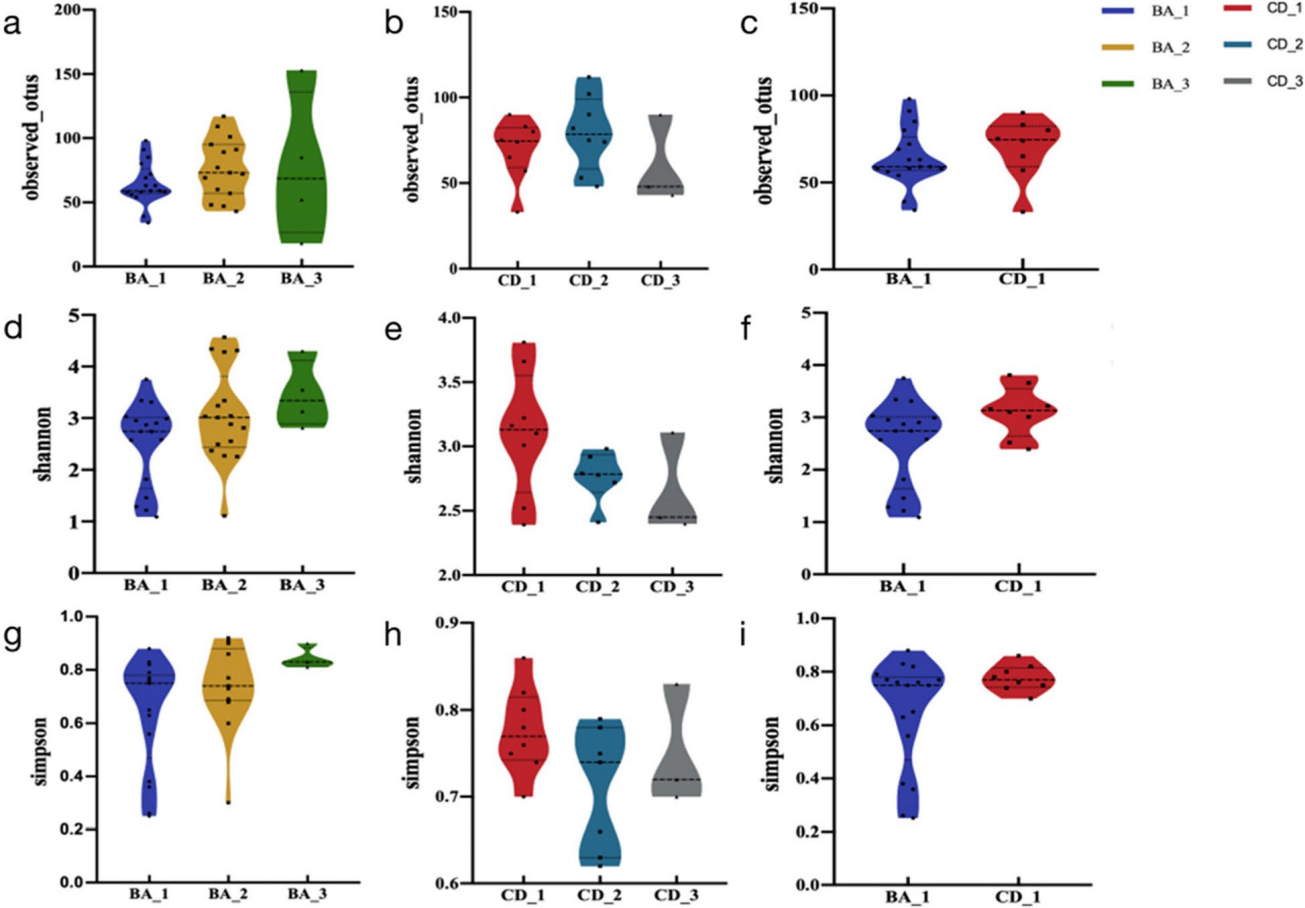
Diversity of intestinal flora in the BA and CD groups and between the two groups before surgery. **a**, **b** There is no significant difference in the number of species in the BA group or the CD group; **c** There is no significant difference in the number of species between the BA group and the CD group before surgery; **d**, **e**, **g**, h There was no difference in the diversity of intestinal flora in the BA group or the CD group; **f**, **i** There was no difference in the diversity of intestinal flora between the BA group and the CD group before surgery

**Fig. 2:**
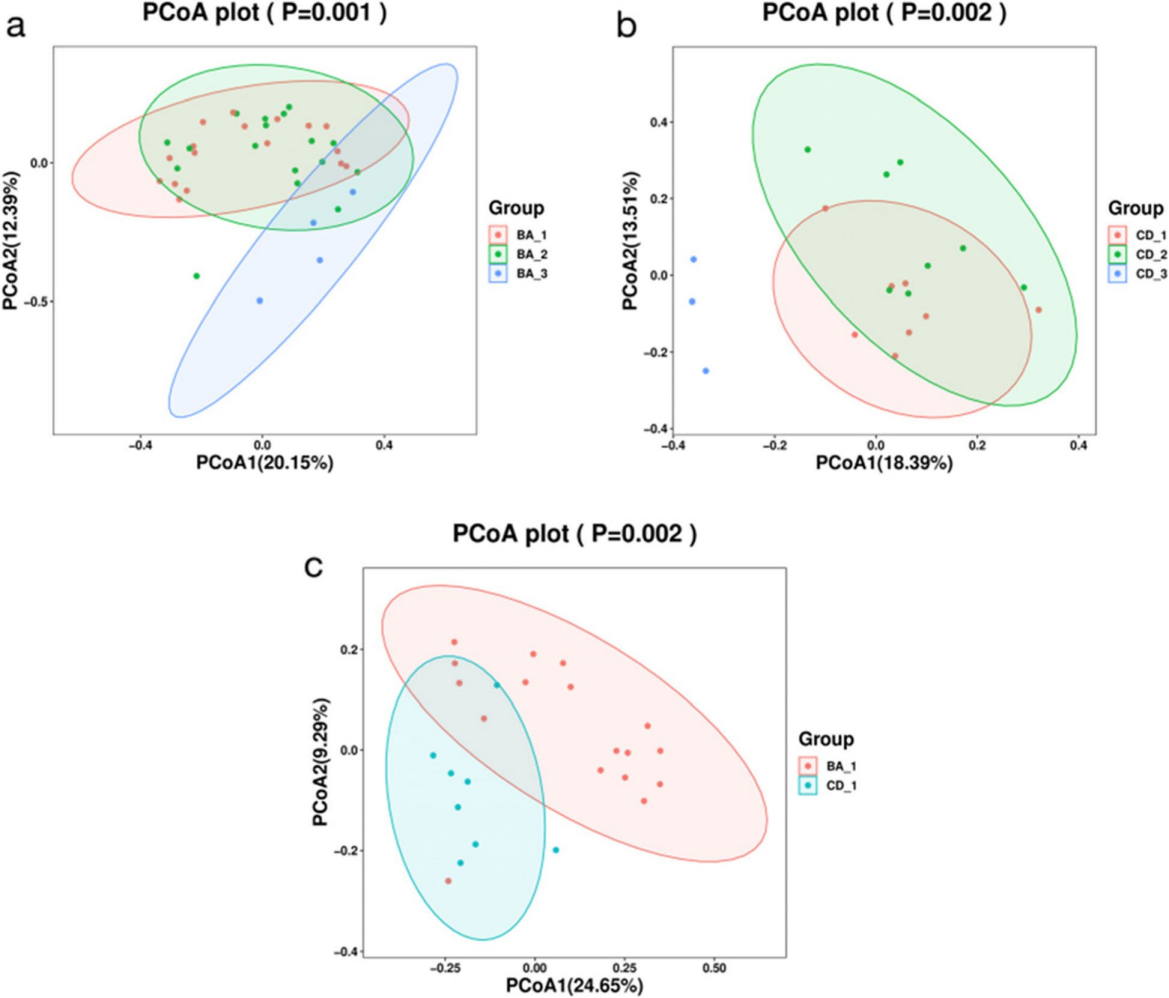
The principal coordinate analysis of the intestinal flora in the BA group and the CD group and between the preoperative groups. **a** The intestinal microbial structure of the BA group was significantly different before and after the operation (*p* = 0.001); **b** The intestinal microbial structure of the CD group was significantly different before and after surgery (*p* = 0.002); **c** The preoperative intestinal microbial structure of the BA group and the CD group was significantly different (*p* = 0.002)

In the Alpha diversity analysis, based on observed_otus, shannon index and simpson index, we found that there was no significant difference in the number and diversity of intestinal microbiota between the BA group and the CD group before and after surgery. This difference was also not apparent between the two preoperative groups (Fig. 1). Beta diversity is demonstrated through principal coordinate analysis (PCoA). Both the differences within the two groups and between the two groups were statistically significant (*p* < 0.05) (Fig. 2).

### Taxonomic analysis

**Fig. 3:**
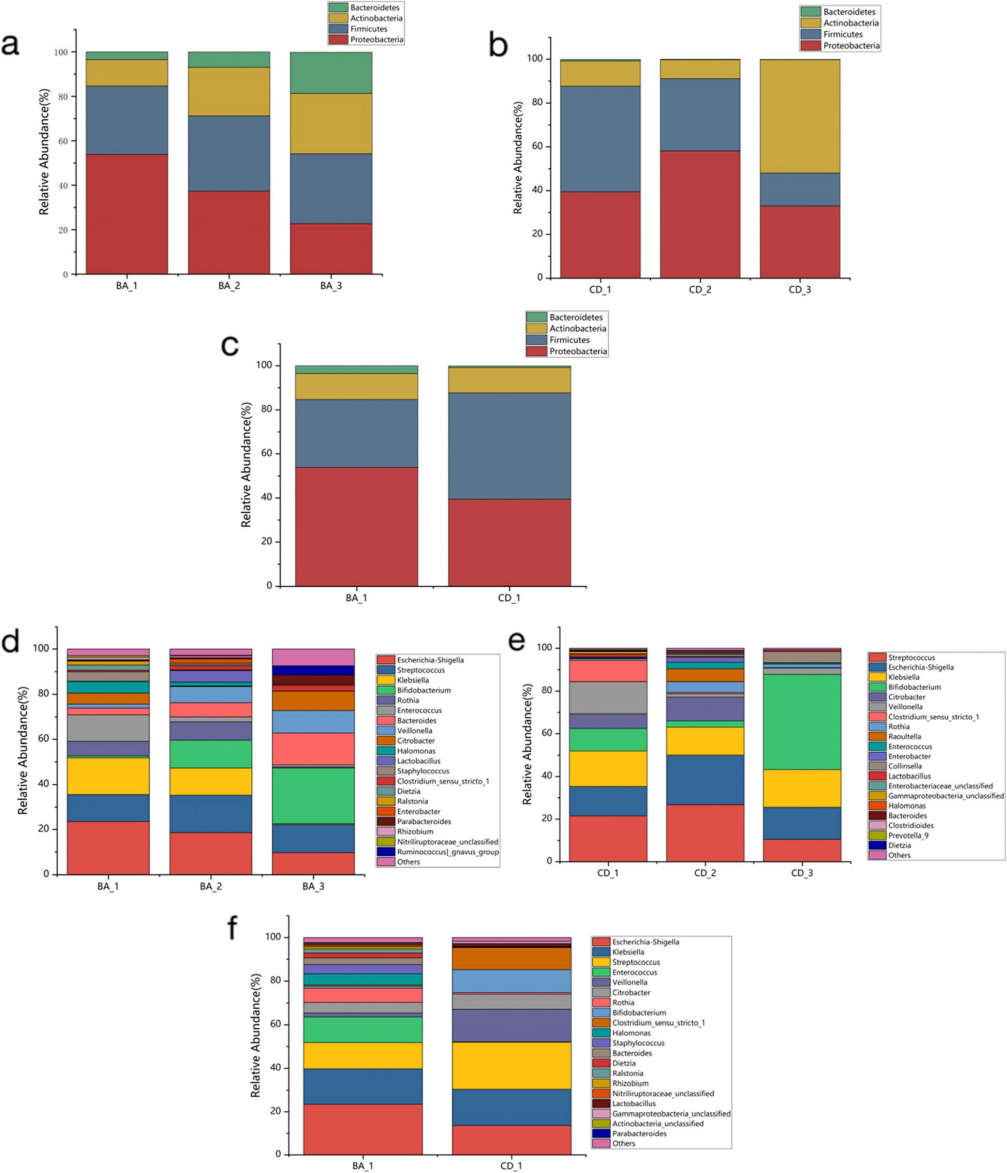
Taxonomic analysis of the intestinal microbiota in the BA and CD groups. **a**, **b**, **c** Taxonomic analysis at the phylum level; **d**, **e**, **f** Taxonomic analysis at the genus level

There are significant differences in the composition of the intestinal microbiota in the BA group and the CD group. At the phylum level, Proteobacteria and Firmicutes have an absolute advantage in the two groups before surgery with the flow of bile, recovery of liver function, and normal serum bilirubin, Bacteroides and Actinobacteria gradually increased in the BA group, and more Actinobacteria in the CD group. At the genus level, pathogenic bacteria such as Shigella, Streptococcus and Klebsiella were found to be significantly enriched in the two preoperative groups, whereas beneficial bacteria such as Bifidobacterium and Lactobacillus were lacking. After surgery, the enrichment of conditional potential pathogens decreased and the proportion of potential probiotics increased. Comparing the preoperative intestinal microbial composition of the two groups, Proteobacteria (53.87%) was significantly enriched than Firmicutes (30.83%), while the CD group represented more than Firmicutes (48.31%). At the genus level, although both groups were increased enteric potential pathogens and reduced potential probiotics, the gut bacterial structure varied significantly. Compared with the CD group, Shigella, Enterococcus, and Rothia were relatively abundant in BA group, and Veillonella, Bifidobacterium, and Clostridium_sensu_stricto_1 were relatively low in the BA group (Fig. 3).

### Significance difference analysis

**Fig. 4:**
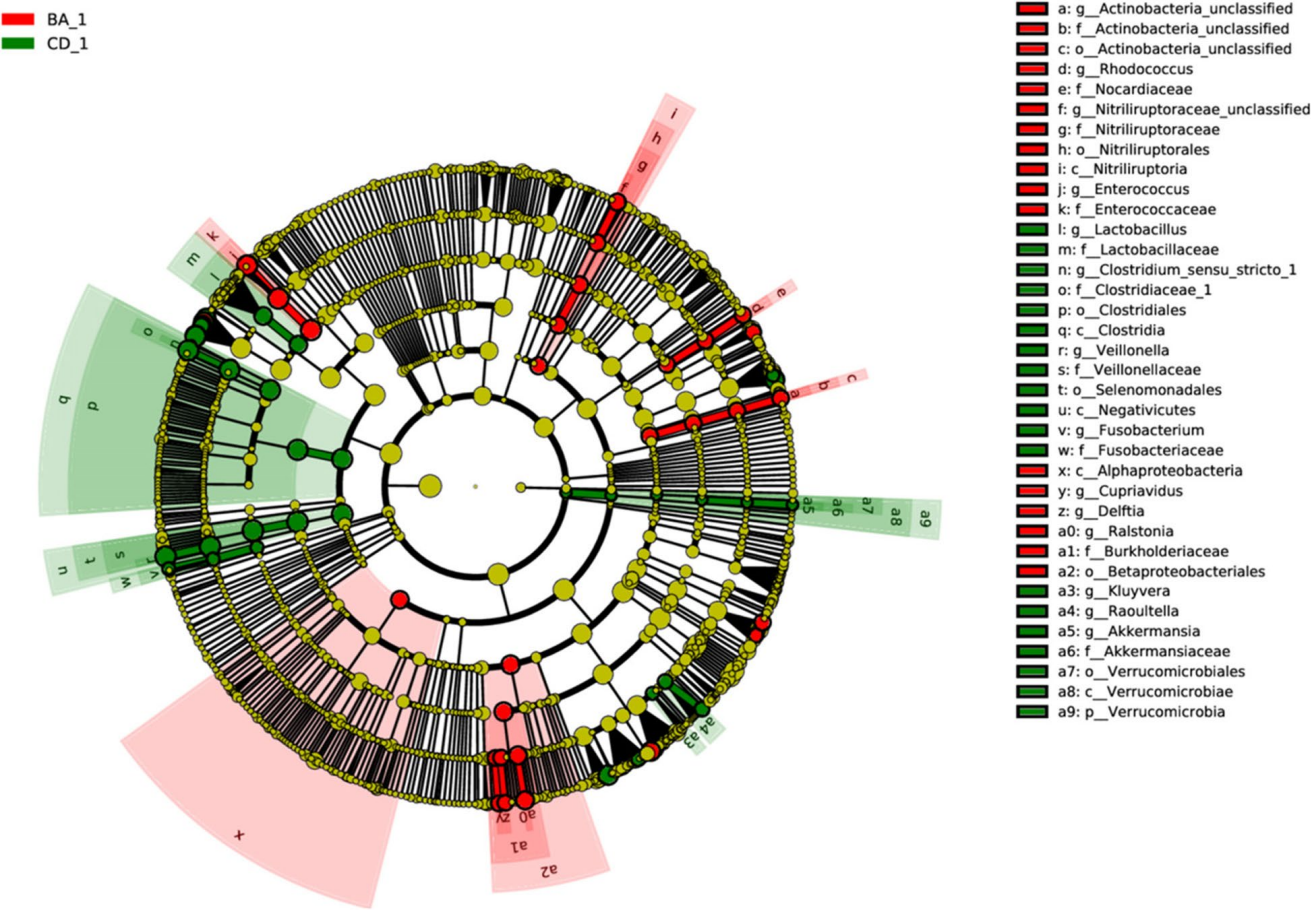
LEfSe (LDA Effect Size) analysis of BA group and CD group. Enterococcus, Ralstonia, Nitriliruptoraceae, Clostridium_sensu_stricto_1, Veillonella, and Lactobacillus have significant differences in relative abundance

The results of LEfSe difference analysis showed a significant enrichment of Enterococcus, Ralstonia, Nitriliruptoraceae in the intestinal microorganisms of infants with biliary atresis (*p* < 0.05). The intestinal microbes of infants with cholestasis were mainly enriched by Veillonella, Clostridium_sensu_stricto_1 and Lactobacillus (*p* < 0.05) (Fig. 4).

### Functional prediction

**Fig. 5:**
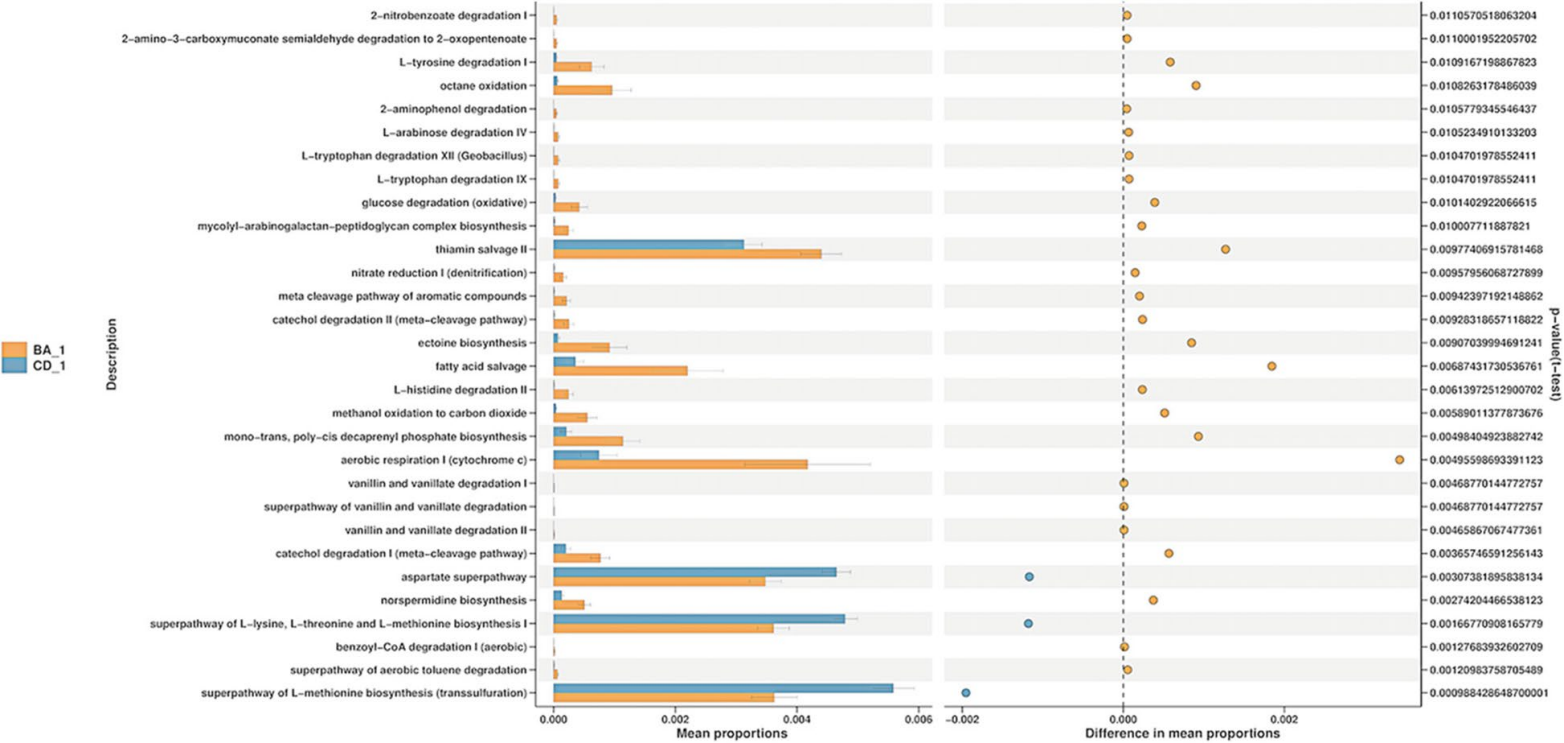
The PICRUSt2 function prediction results of the intestinal flora of the BA group and the CD group. The abundance data in the functional database shown in the results are statistically significantly different functions (95% confidence interval), and it can be preliminarily inferred that the flora is related to the related functions in the figure

Based on the PICRUSt2 function prediction results, STAMP differences between groups were analyzed by t-test, and the distribution of intestinal microbes pathways between the two groups was obtained. Rich functional categories in BA group are “L − tyrosine degradation I” (*p* = 0.011), “L − histidine degradation II” (*p* = 0.006), “glucose degradation” (*p* = 0.010), “aerobic respiratory I” (*p* = 0.005), “catechol degradation I”(*p* = 0.004), “thiamin salvage II”(*p* = 0.010), “ectoine biosynthesis”(*p* = 0.009), “fatty acid salvage”(*p* = 0.007); The rich functional categories in CD group are “aspartate superpathway” (*p* = 0.003), “superpathway of L − lysine, L − threonine and L − methionine biosynthesis I” (*p* = 0.002), “superpathway of L − methionine biosynthesis” (*p* = 0.001) et al. (Fig. 5).

## Discussion

In this study, the differences in preoperative gut microbes with biliary atresia and non-biliary atretic cholestasis were clarified. In addition, by longitudinal comparisons, changes in gut microbes were observed before and after surgery. It is understood that this is the first study of the difference in intestinal microbiota between biliary atresia and cholestasis before surgery.

Between the two groups we observed that although there was no significant preoperative difference between the two groups in terms of species diversity, a trend of rich diversity in the CD group compared with the BA group was seen. In the gastrointestinal tract, disruption of the intestinal and hepatic circulation of bile acids can cause inflammation. As bile acids further remained within hepatocytes, hepatocyte DNA damage, apoptosis, and inflammatory gradually emerged [24]. Inflammation leads to disruption of intestinal microecology and disordered microbiota diversity. Compared with the incompletely obstructed biliary tract in the CD group, the bile outflow pathway in the BA group was completely blocked, causing more obvious damage. In terms of structure of the intestinal microbiota, the two preoperative groups varied significantly, indicating that the microbial types contained in the two diseases were significantly different, and also laying the foundation for finding specific differential bacteria later. Otherwise, the way of delivery, gestational age, growth environment, feeding patterns will also affect the composition of their intestinal microbiota [25]. After the biliary obstruction was completely relieved by surgery, there was still no significant difference in the diversity of the intestinal microbiota compared with that before the operation, but the structure of the microbiota was significatly changed (*p* < 0.01). It is worth mentioning that the two groups showed the opposite trend in species diversity before and after surgery: the BA group increased and became stable, but slightly decreased in the CD group. Combined with the taxonomic analysis, it is found that perhaps because of the smooth excretion of bile, the original relatively large potential pathogens decreased, and some potential probiotics occupy the advantage.

About 10^11−12^bacteria colonize in the human colon, of which over 90% belong to Bacterioides and Firmicutes. In the intestinal microbiota of infant with cholestasis, we saw a significantly reduced relative abundance of Bacterioides and Firmicutes (BA group, 34.31%; CD group, 50.00%) and a marked increase in the proportion of Proteobacteria (BA group, 58.87%; CD group, 39.44%). Proteobacteria has been found to increase in certain metabolic diseases (such as obesity, diabetes, alcoholic hepatitis, cardiovascular disease, etc.), inflammatory phase of the disease, and inflammation-related bowel disease [26]. Shin et al. [27] proposed that Proteobacteria may act as a "microbial feature" of the disease. Furthermore, the relative abundance of Proteobacteria in the BA group and lower richness of Bacterioides and Firmicutes compared to the CD group, suggesting more severe gut microbial dysbiosis in the BA group.

In order to study the intestinal microorganisms of the two groups at the genus level, we analyzed the differences of fecal flora between the two groups before operation. The main purpose is to find the species with significant differences in the relative abundance of intestinal microorganisms between the two groups. We found a total of 14 genera as differentially abundant. Enterococcus, Ralstonia and Nitriliruptoraceae were the most significantly enriched in the BA group, and Clostridium_sensu_stricto_1, Veillonella and Lactobacillus were significantly enriched in the CD group. Enterococcus is Gram-positive cocci, an important pathogen of nosocomial infection. Enterococcus has been thought to be associated with many diseases in humans, causing not only urinary tract infections, soft tissue infections but also life-threatening celiac infections, septicemia, endocarditis and meningitis. Guo et al. [28] also reported a significant enrichment of Enterococcus in infantile cholestasis. Ralstonia is a clinically rare opportunistic pathogen that belongs to non-fermented Gram-negative bacteria. It is thought to be associated with serious disease, pathogenic mainly in immunocompromised hosts, and can cause sepsis and infected in tissues such as the lung and brain [29, 30]. Previously, Ralstonia has rarely been reported in infant and has not been detected in infant hepatobiliary disease. Nitriliruptoraceae belongs to the Actinobacteria, which is currently poorly known in humans for the first time found in human disease. Clostridium_sensu_stricto_1 is a Gram-positive bacterium and is a species of Clostridium. The relative abundance of Clostridium_sensu _stricto_1 was significantly increased in the gut microecological imbalance. Ji et al. [31] found significant enrichment of Clostridium_sensu_stricto_1 in a model of necrotic enterocolitis in neonatal mice. It was also found to be significantly enriched in IBS syndrome [32] and leukemia [33]. This is the first report of Clostridium_sensu_stricto_1 in cholestasis. Veillonella is a gram-negative anaerobic micrococcus that is a normal flora distributed in the oral cavity, pharynx, respiratory tract, and digestive tract. Wang et al. [16] indicated that Veillonella is significantly increased in the intestinal microbiota of infants with cholestasis, which may be related to the decrease of bile acid in the intestine. Lactobacillus is widely distributed in nature, and some strains are one of the normal flora in the oral, gut and vagina of human and animals, rarely pathogenic and basically harmless to people. Lactobacillus parasitic to the gut and vagina is protective against the body. It has been found that Lactobacillus, for carrying the *bsh* gene, can enhance bile salt hydrolase activity, thus contributing to bile resistance and has a cholesterol reduction effect. Lactobacillus bile salt hydrolase substrate specificity is able to control bacterial adaptability and host colonization [34]. These differential microbiota may become potential biomarkers for identifying biliary and cholestasis. With the excretion of bile acids, potential probiotics were found to increase significantly than preoperative, suggesting that early postoperative supplementation of potential probiotics may help in the recovery of intestinal microecology.

Gut microbiota performs many important functions in the host, with the establishment of a real symbiosis. These functions include metabolism and synthesis of nutrients, notably vitamin K and B group vitamins, tropism on the mucosa, drugs and toxins metabolism, and barrier functions [26]. Guo et al. [28] found that compared with normal infants, the rich functional categories in the intestinal flora of infants with cholestasis include lipid metabolism, glycan biosynthesis and metabolism, and xenobiotic biodegradation and metabolism. In contrast, amino acid metabolism and nucleotide metabolism are reduced [35]. In this study, based on the results of PICRUSt2 function prediction, we found that the gut microbial function in BA group is mainly concentrated in the process of energy release such as amino-acid degradation, glucose degradation and aerobic respiration, while CD group function is concentrated in the biosynthesis of amino-acids, mainly for energy consumption. Differences in gut microbial function in the BA group and CD groups suggest a distinct gut microbiota composition for the two diseases. Inteintestinal microbes regulate multiple host metabolism and immune processes, suggesting that different dysregulation patterns may exist in both disorders, which may be what is our next step.

A comprehensive analysis of the intestinal microbiota before and after surgery in both longitudinal and lateral aspects has found that the preoperative intestinal microecological imbalance of biliary atresia may be more serious. We also showed the change process of the intestinal microbiota before and after the operation of the two diseases, providing a new perspective for the prognosis of the operation. More importantly, this study has discovered possible biomarkers for biliary atresia and non-biliary atretic cholestasis. This new research field of intestinal microbiota provides a possible new method for the identification and diagnosis of these two diseases. This is the first study to distinguish biliary atresia from cholestasis from a gut microbe perspective. Despite these advantages, we still have some limitations, such as: 1. the changes in intestinal bile acids were not studied; 2. the impact of antibiotics on the postoperative intestinal microbiota can not be estimated; 3. based on a small sample size and need further study to confirm their utility as potential diagnostic/diagnostic indicators; 4. postoperative observation time is less. These limitations are the focus that we will study next.

## Conclusion

The gut microbiota composition differs between biliary atresia and non-biliary atresia cholestasis. Preoperative potential pathogenic bacteria increased and potential probiotic bacteria decreased in the two groups, and the gradual recovery of this abnormal increase or decrease after surgery may indicate the prognosis of surgery. Enterococcus, Ralstonia and Nitriliruptoraceae, Clostridium_sensu_stricto_1, Veillonella and Lactobacillus may be biomarkers for the differential and diagnosis of biliary atresia and non-biliary atresia cholestasis.

## Data Availability

The datasets generated and analysed during the current study are available in the MetaboLights repository, https://www.ebi.ac.uk/metabolights/MTBLS5668.

## Abbreviations

BA: Biliary atresia
CD: Cholestasis disease
PFIC: Progressive familial intrahepatic cholestasis
AS: Alagille syndrome
KPE: Kasai portoenterostomy
ALT: Alanine aminotransferase
AST: Alanine aminotransferase
TBIL: Total bilirubin
DBIL: Direct Bilirubin
PCoA: Principal coordinate analysis
